# Formation of Maillard Reaction Products in Aged Sorghum Vinegar during Ageing and Protective Effects of Pure Vinegar Melanoidin Against CCl_4_-Induced Rat Hepatic Damage

**DOI:** 10.17113/ftb.61.01.23.7537

**Published:** 2023-03

**Authors:** Xiaomin Tang, Xiaoyu Yin, Majida Al-Wraikat, Yaqiong Zhang, Saiping Zhou, Yingxue Tang, Yanyan Zhang, Junfeng Fan

**Affiliations:** 1 Department of Food Science and Engineering, College of Biological Sciences and Technology, Beijing Key Laboratory of Forest Food Processing and Safety, Beijing Forestry University, 35 Qinghua Road, Haidian District, 100083 Beijing, PR China; 2 College of Food Science and Engineering, Beijing University of Agriculture, 7 Beinong Road, Changping District, 102206 Beijing, PR China

**Keywords:** food safety, 5-HMF, advanced glycation end products, hepatoprotective effect, oxidative damage, reactive oxygen species

## Abstract

**Research background:**

The processing method generally affects the toxicity and biological activity of aged sorghum vinegar. This study investigates the changes in the intermediate Maillard reaction products of sorghum vinegar during ageing and the *in vivo* hepatoprotective effects of pure melanoidin obtained from it.

**Experimental approach:**

High-performance liquid chromatography (HPLC) and fluorescence spectrophotometry were utilized to quantify intermediate Maillard reaction products. The CCl_4_-induced liver damage in rats was used to evaluate the protective role of pure melanoidin in rat liver.

**Results and conclusions:**

Compared with the initial concentration, the 18-month ageing process caused a 1.2- to 3.3-fold increase in the concentrations of intermediate Maillard reaction products, *i.e.* 5-hydroxymethylfurfural (HMF), 5-methylfurfural (MF), methyglyoxal (MGO), glyoxal (GO) and advanced glycation end products (AGEs). The concentrations of HMF in the aged sorghum vinegar were 6.1-fold higher than the 450 μM limit standard for honey, implying the need for shortening the ageing of the vinegar in practice for safety concerns. Pure melanoidin (*M*_r_>3.5 kDa) demonstrated significant protective effects against CCl_4_-induced rat liver damage, as evidenced by normalized serum biochemical parameters (transaminases and total bilirubin), suppressing hepatic lipid peroxidation and reactive oxygen species, as well as increasing glutathione amount and restoring antioxidant enzyme activities. Histopathological analysis revealed that melanoidin in vinegar reduced cell infiltration and vacuolar hepatocyte necrosis in rat liver. The findings demonstrated that a shortened ageing process should be considered in practice to ensure the safety of aged sorghum vinegar. Vinegar melanoidin is a potential alternative for the prevention of hepatic oxidative damage.

**Novelty and scientific contribution:**

This study demonstrates that the manufacturing process had a profound influence on the generation of vinegar intermediate Maillard reaction products. In particular, it revealed the *in vivo* hepatoprotective effect of pure melanoidin from aged sorghum vinegar, and provides insight into the *in vivo* biological activity of melanoidin.

## INTRODUCTION

Maillard reaction is a non-enzymatic reaction commonly occurring between carbonyl compounds (reducing sugar) and amino compounds (protein, peptides and amino acids) during the processing and storage of foods, such as soy sauce, oyster sauce, bread and vinegar, which enhance the flavour and colour of food. Maillard reaction products represent a series of compounds, including key compounds such as 5-hydroxymethylfurfural (HMF), 5-methyfurfural (MF), methylglyoxal (MGO), glyoxal (GO), advanced glycation end products (AGEs) and melanoidins. Along with the increasing consumer awareness of food nutrition and safety in recent years, studies are increasingly focusing on the healthy and harmful effects of Maillard reaction products on human body. For example, the products such as HMF, MF, MGO, GO, AGEs and acrylamide have been implicated in ageing and chronic degenerative diseases, even diabetes and cancers (*1*), while low-molecular-mass compounds and melanoidins in Maillard reaction products are associated with numerous biological activities, such as antibacterial, antioxidant, anti-inflammatory and immunomodulatory effects (*2*). Currently, the amounts of Maillard reaction products in final products are determined in several foods including soy sauce, oyster sauce and rice vinegar, albeit rarely (*1*). Because they are generally formed via a series of reactions including oxidation, dehydration, cyclization and polymerization during food processing and storage under heating or room temperature, insight into the Maillard reaction product processing, ageing and storage is very important to resolve concerns of food safety and nutrition.

**Fig. S1:**
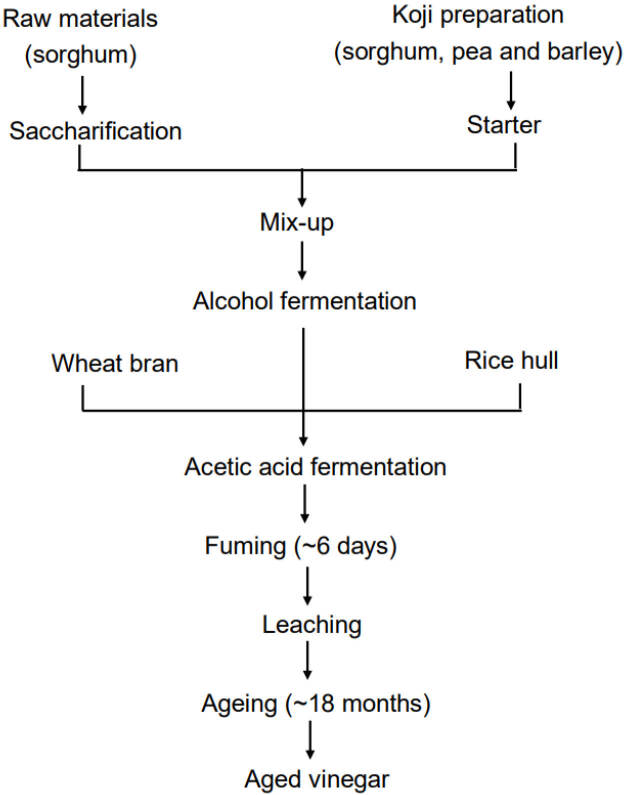
Flowchart for the manufacturing of aged sorghum vinegar

Aged sorghum vinegar is common seasoning used traditionally in north China. Pen Ts’ao Kang Mu, the *materia medica* compiled by Li Shi-Zhen (1518-1593), describes the use of aged sorghum vinegar to dispel blood stagnation, treat jaundice and foster liver (*3*). It has therefore been used as a common seasoning to treat liver diseases, carbuncles and inflammation. Recent studies have shown that vinegar exhibits many pharmacological activities, including hepatoprotection as well as regulation of blood glucose, blood pressure and antithrombotic activities (*4*-*8*). It is believed that the nutritional, pharmaceutical and even toxic properties of foods and common seasoning correlate closely with those of raw materials and processing methods. Sorghum is the main ingredient (around a half of the primary material) of koji, which also contains wheat bran, oat, Tartary buckwheat and pea as fermentation starter. The final vinegar product contains many ingredients, and their amount affects its quality (*9*). Furthermore, the production of aged sorghum vinegar also entails complex solid-state fermentation (Fig. S1), especially including saccharification of raw materials, alcohol and acetic fermentation, fuming, leaching and ageing. The 6-day fuming (heating at ~100 °C) increases the concentrations of organic acids, flavour volatiles and undoubtedly Maillard reaction products due to a series of non-enzymatic browning reactions in the vinegar. The ageing of vinegar for more than 18 months involves defrosting and rapid evaporation of moisture during the hot summer. The final vinegar is highly condensed, resulting in enhanced flavour and colour. Therefore, Maillard reaction products are generated due to further chemical reactions among low-molecular-mass compounds, such as sugars, amino acids and polyphenols under temperature variation and highly condensed acidic suspension. Currently, most of the studies focus on the role of fuming on the biological activities of melanoidins in aged sorghum vinegar (*1*, *10*, *11*), while the formation of Maillard reaction products during sorghum vinegar ageing has yet to be elucidated.

Many factors, such as virus infection, excessive intake of alcohol and chemical drugs, cause liver damage. Oxidative stress induced by reactive oxygen species (ROS) is a major mechanism underlying liver damage (*12*). The ability of the antioxidants to terminate free radical formation and to inhibit subsequent lipid peroxidation in the liver is crucial for their hepatoprotective activity. Phenolic extracts derived from aged sorghum vinegar showed antioxidant activity in ageing mice exposed to ^60^Co γ-ray irradiation and hepatoprotective efficacy against oxidative damage *in vitro* and *in vivo* (*8*, *13*). Melanoidins derived from other food sources act as radical scavengers, metal chelators and inhibitors of low-density lipoprotein (LDL) oxidation *in vitro* (*14*). As one of the major functional ingredients in vinegar, melanoidins also exhibit antibacterial, hypolipidaemic, anti-angiotensin-converting enzyme (ACE) activity, and in particular, antioxidant activity (*15*, *16*). However, the currently available studies investigating the antioxidant activity of melanoidins were only conducted *in vitro* (*10*, *17*). No *in vivo* studies evaluated its hepatoprotective activity. In particular, the bioactivity of vinegar melanoidins is mostly attributed to the non-covalently bound phenolics. The action of pure melanoidins has yet to be elucidated.

Given the high possibility of adverse or beneficial Maillard reaction products, the study evaluated the progress and stability of the major Maillard reaction products during the ageing of sorghum vinegar. Melanoidins, one of the major Maillard reaction products, were also isolated from aged vinegar and characterized. Their potential protective effect against CCl_4_-induced hepatic oxidative damage was further evaluated in a rat model to elucidate the biological activity of pure melanoidins. In addition, the possible protective mechanism of melanoidins from the aged sorghum vinegar in liver oxidative damage was also elucidated in mice.

## MATERIALS AND METHODS

### Materials

Glucose standard and acetic acid were purchased from Tianjin Yongda (Tianjin, PR China). 5-Hydroxymethylfurfural (HMF), 5-methylfurfural (MF), methyglyoxal (MGO; 40% solution in water), glyoxal (GO; 40% solution in water), *o*-phenylenediamine, 2,2-diphenyl-1-picrylhydrazyl (DPPH), 2,7-dichlorofluorescin, Folin-Ciocalteu reagent and gallic acid were purchased from Sigma-Aldrich, Merck (St. Louis, MO, USA). Test kits used for the determination of total superoxide dismutase (SOD), glutathione peroxidase (GPx), catalase (CAT), malondialdehyde (MDA) and serum biochemical markers (alanine transaminase (ALT) aspartate transaminase (AST), alkaline phosphatase (ALP) and total bilirubin) were purchased from Nanjing Jiancheng Bioengineering Institute (Nanjing, PR China). All reagents were of analytical grade.

### Production and chemical analysis of vinegar

Aged sorghum vinegar was produced and supplied by Shanxi Ziyauan Microorganism R&D Co., Ltd. (Taiyuan, PR China). It is made from a mixture of sorghum (450 g/kg), wheat bran (140 g/kg), rice bran (140 g/kg) and oat and peas (270 g/kg), which is processed into the aged vinegar through koji fermentation, starch saccharification, alcohol fermentation, acetic acid fermentation, fuming, leaching and ageing (Fig. S1). After leaching, samples were collected every six months until the 18th month of ageing in an open vat.

Moisture and ash were estimated in a vacuum drying oven (DZF; Longyue, Shanghai, PR China) and muffle furnace (SX-G07103; Zhonghuan, Tianjin, PR China) according to a previous study (*9*). Protein content was calculated from nitrogen by multiplying with 6.25 using a Kjeldahl apparatus (KDY-9830; Ruibang Xingye, Beijing, PR China) based on a previous method (*18*). The carbohydrate content was analyzed according to a previous study (*9*). The pH was measured with a digital pH meter (BPP-7800; BELL, Dalian, PR China). Titratable acidity was determined in g of acetic acid per 100 mL using the standard method (*9*).

### The analysis of Maillard reaction products

Maillard reaction products including HMF, MF, MGO, GO and AGEs were analyzed according to a previously reported method (*1*). To determine HMF and MF, the sample was diluted 10-fold with methanol (*V*/*V*), followed by vortexing for 10 s (HY-1; Leici, Shanghai, PR China), ultrasonication for 15 min (JP-070S; Skymen, Guangdong, PR China), and centrifugation at 8000×*g* for 15 min (GL-20G-II; Anting, Shanghai, PR China). After filtration through a 0.45*-*μm membrane, the supernatant was analyzed using a Shimadzu LC-20ATA HPLC system (Shimadzu, Tokyo, Japan) equipped with a diode array detector and a GRACE® VYDAC® C18 column (5 μm×4.6 mm×25 mm; W. R. Grace, Cambridge, MA, USA). The mobile phase was composed of acetonitrile (ACN) and water with 0.1% formic acid and gradient elution from 5–70% ACN in 33 min at 1 mL/min was performed. The equation and correlation coefficients of HMF and MF standard curves, respectively, were as follows:

y=168517x–6734, r^2^=0.9995 /1/

y=276729x–3437.5, r^2^=0.9999 /2/

The concentrations of MGO and GO were analyzed by diluting the sample 10-fold with deionized water (*V*/*V*). An aliquot of 1 mL of diluted vinegar was reacted with 5 μL *o*-phenylenediamine (500 mM) at 37 °C for 2 h. After stopping the reaction with cold perchloric acid, the solution was centrifuged and filtered as mentioned above. The filtrate was injected into the Shimadzu HPLC system (Shimadzu). The mobile phase contained ACN and 0.3% acetic acid in water, with gradient elution from 35 to 555% ACN in 25 min at 1 mL/min. The equation and correlation coefficients of the standard curves for MGO and GO, respectively, were as follows:

y=14685x–12621, r^2^=0.999 /3/

y=8340x–1856.7, r^2^=0.9996 /4/

To determine the AGEs and melanoidins, the sample was diluted 100-fold with deionized water (*V*/*V*) and ultrasonically treated for 30 min, followed by centrifugation (8000×*g* for 15 min). The fluorescence intensity of the supernatant was determined at an excitation/emission wavelength of 355/405 nm with a Victor X4 fluorescence spectrophotometer (PerkinElmer, Waltham, MA, USA) to assay the AGEs (*1*). The concentration of melanoidins in the supernatant were determined at 420 nm with a PE lambda 750 UV-visible spectrometer (PerkinElmer) and the raw melanoidin in the sample was calculated using the following equation:

*γ*=*A·V*_1_/(0.269*V*_2_·10) /5/

where *γ* is the concentration of melanoidin in the sample (mg/mL), *A* is the absorbance of the sample at 420 nm, *V*_1_ represents constant volume (mL), *V*_2_ refers to the volume of the sample and 0.269 is the absorbance of 0.1 mg/mL melanoidin at 420 nm.

### Preparation and physicochemical analysis of vinegar melanoidins

The melanoidins present in the 18-month aged vinegar were analyzed as described before (*19*). Briefly, after a 10-fold dilution with deionized water, the diluted vinegar was filtered and the filtrate was further ultrafiltered using a membrane (*M*_r_=3.5 kDa) to yield a high-molecular-mass fraction of melanoidins (*M*_r_>3.5 kDa). To eliminate non-covalently bound compounds from the melanoidin skeleton, NaCl was added to the high-molecular-mass fraction solution (50 mg/mL) until the concentration of NaCl reached 2 M. The solution was incubated overnight and subjected to ultrafiltration as stated above. The resultant retentate was lyophilized and referred to as vinegar melanoidin.

Wavelength spectra between 190 and 700 nm of vinegar melanoidins (0.25 mg/mL) were recorded using a Mini-1240 UV/Vis spectrophotometer (Shimadzu). The *M*_r_ distribution of vinegar melanoidins was determined by high-performance gel permeation chromatography (HPGPC) using a Shodex OHpak SB-806M HQ column (Showa Denko K.K., Tokyo, Japan), equipped with both a refractive index detector (RID) (JKY/S; Beijing Midwest Yuanda, Beijing, PR China) and a multi-angle laser scattering detector (MALSD) (DAWN HELEOS-II; Wyatt Technology, Santa Barbara, CA, USA) as described previously (*20*). Concentrations of protein, total carbohydrate and melanoidins in vinegar were determined using the method described above. Total phenolic content of vinegar melanoidins was determined based on Folin-Ciocalteu method (*21*) with gallic acid as the standard. HMF, MF, MGO, GO and AGEs in vinegar melanoidins were analyzed according to the method above.

^1^H and ^13^C NMR spectra were recorded with a Bruker Advance 500 spectrometer (Bruker Optics, Ettlingen, Germany) using 20 mg/mL sample in D_2_O (0.5 mL). To obtain FTIR spectra, sample powders were blended with KBr and pelleted, followed by 70 scans from 4000 to 400 cm^-1^ at a resolution of 4 cm^-1^ using a Bruker Vertex 70 (Bruker Optics) at room temperature.

### In vitro antioxidant activities

DPPH radical scavenging activity was determined as described by Liu *et al.* (*22*) with Trolox as standard. Briefly, appropriately diluted melanoidins (100 μL) were mixed with 100 μL of 200 μM DPPH solution in 0.1 M 2-(*N*-morpholino)ethanesulfonic acid (MES) buffer, pH=6.0 (Sigma-Aldrich, Merck) containing 80% ethanol. The mixture was left at room temperature for 20 min. The absorbance of the resulting solution was then measured at 517 nm using a plate reader (model 550; Bio-Rad Laboratories, Tokyo, Japan). The antioxidant activity was expressed in mg Trolox equivalents per g vinegar melanoidins.

Oxygen radical absorbance capacity (ORAC) assay was performed based on the method described by Liu *et al.* (*22*) The final reaction solution (200 μL) was composed of fluorescein (120 μL, 70 nM), ABAP (60 μL, 12 mM), appropriately diluted samples (20 μL) and phosphate buffer (75 μM, pH=7.4). Trolox was used as a standard and ORAC results were expressed in μM Trolox equivalents per g vinegar melanoidins.

### In vivo analysis of hepatoprotective activity

Sprague-Dawley rats weighing 155–190 g were housed in cages with conventional stainless steel wire bottom and maintained at controlled temperature ((22±1) °C) and light/dark cycle 12 h/12 h. They were fed with a standard laboratory diet and water *ad libitum*. The experiment was approved by the Institutional Animal Care and Use Committee of Beijing Forestry University (#2018PN016).

The animals were randomly divided into six groups each consisting of ten rats. Group 1 was administered corn oil and served as control. The remaining five groups were gavaged with carbon tetrachloride suspended in corn oil (200 g/kg of CCl_4_ in corn oil; 0.5 mL per kg of body mass (bm)) twice a week (on Tuesday and Friday) for 6 weeks to induce chronic reversible cirrhosis. The effective dose of aged vinegar was 1.5–6 mL per kg of bm as demonstrated in our previous study investigating the hepatoprotective effects of aged vinegar in rats (*13*). The yield of vinegar melanoidins was 61.5 mg/mL in the present study. Hence, 400 mg/kg and subsequently lower doses (200 and 100 mg/kg) of vinegar melanoidins were selected for the experiments. In addition to the CCl_4_ suspension, groups 3, 4 and 5 were treated with vinegar melanoidins (100, 200 and 400 mg per kg of bm, respectively) while group 6 received 100 mg per kg of bm silymarin. The rats were fed both vinegar melanoidins and silymarin five times a week (on Monday, Wednesday, Thursday, Saturday and Sunday). Blood samples were collected from the tail vein of the rats to a tube containing heparin (10 U/mL) before the first CCl_4_ treatment and at the end of the sixth week. At the end of the sixth week, the animals were anesthetized using ether and sacrificed. Serum was separated from the blood *via* centrifugation (TD5A-WS, Xiangyi; Changsha, Hunan, PR China) at 3000×*g* for 10 min and used to measure various biochemical parameters. The liver removed from rats was immediately harvested after blood collection, minced in an ice-cold solution containing 0.25 M sucrose, 10 mM Tris(hydroxymethyl)aminomethane base and 0.5 mM ethylenediamine tetraacetic acid (EDTA) (pH=7.4, 1:9 *m*/*V*), and finally homogenized for 15 min using a Polytron homogenizer (model T25S1; IKA Labortechnik, Staufen, Germany). The homogenate was centrifuged at 2000×*g* and 4 °C for 20 min. The supernatant was used immediately to measure the chemical and enzyme activity.

Biochemical parameters including AST, ALT, ALP and total bilirubin were measured using test kits (Nanjing Jiancheng Bioengineering Institute, Nanjing, PR China) (*23*, *24*). Glutathione in the supernatant of 100 mg/mL liver homogenate prepared in phosphate-buffered saline (PBS) was measured according to Moron *et al.* (*25*). Lipid peroxidation was estimated based on the formation of MDA in liver homogenates (*26*). The results were expressed in nmol MAD/mg protein.

The total activities of SOD (EC1.15.1.1), GPx (EC1.11.1.9) and CAT were determined using the corresponding test kits. The total SOD activity was determined using the method reported by Carpo *et al.* (*27*) One unit of SOD activity was defined as the amount of enzyme required to inhibit the reduction of cytochrome *c* by 50%. The activity of GPx was determined by measuring the amount of nicotinamide adenine dinucleotide phosphate (NADPH) utilized during the reduction of hydrogen peroxide and other organic hydroperoxides (ROOH) by glutathione (*28*). CAT activity was determined by monitoring the decomposition of hydrogen peroxide at 240 nm (*29*) and was expressed in units per gram of protein.

To determine hepatic ROS, rat livers were washed with D-Hank’s balanced salt solution (Phygene, Wuhan, Hubei, PR China) and cut into small pieces (1 mm×1 mm) with a sterilized surgical bistoury to obtain the liver cell suspension. After two washes with D-Hank’s solution containing 5 mg/mL EDTA, the liver pieces were hydrolyzed with collagenase (1 g/kg) and trypsin (5 g/kg) at 37 °C for 40 min, followed by squeezing of the hepatocytes from the hydrolysate through a 400-mesh nylon mesh. The hepatocytes were centrifuged at 20×*g* for 5 min, and the precipitate was washed twice with PBS. The liver cell suspension (5·10^5^ cell/mL) was obtained by adding RPMI-1640 containing 100 mg/mL bovine serum to the washed precipitate. To quantify ROS, the liver cells were plated in 24-well multi-well plates at 5·10^5^ cell/well, followed by the addition of 10 µM DCFH to each well (*30*). The plates were incubated for 30 min at 37 °C. Fluorescence of the liver cells was recorded at an excitation wavelength of 485 nm and an emission wavelength of 530 nm (Synergy H4 Hybrid; Aosailite Scientific Co., Beijing, PR China).

To perform histological analysis (transmission electron microscopy of liver sections), rat liver fragments were fixed in 100 mg/mL neutral buffered formalin and embedded in paraffin (*31*, *32*). Sections of 5-6 μm thick rat livers were stained with hematoxylin and eosin, followed by histological analysis using a compound microscope (Leica DMIL; Leica Microsystems, Wetzlar, Germany). Images of the rat livers were captured using a Leica DFC 208 CCD camera (Leica Microsystems, Wetzlar, Germany).

### Statistical analysis

Each experiment was performed in triplicate. The results were expressed as mean value±standard deviation. Statistical analyses were conducted using Origin v. 2021 (*33*) and SPSS Statistics v. 26.0 (*34*). Statistical comparisons were made *via* one-way analysis of variance, followed by Duncan’s multiple comparison. Differences were significant when p<0.05.

## RESULTS AND DISCUSSION

### Changes in the proximate composition of vinegar during ageing

The ageing results in thickening of vinegar, with high acidity, mellow flavour and reddish-brown colour by evaporation in the summer and defrosting in the winter. In this study, the vinegar volume was reduced to approx. two-fifths of the original volume after ageing for 18 months, based on the decrease in moisture from 873.5 to 676.5 g/L (Table 1). During the entire ageing process, the pH showed a slight decrease but basically remained constant, while the titratable acidity showed a significant increase from 45.5 to 83.4 g/L (wet basis), which indicated that the vinegar became concentrated by rapid evaporation of water during ageing. However, when the titratable acidity was calculated based on the dry basis, it actually decreased from 359.7 mg/g at the beginning of ageing to 257.8 mg/g at the end (data not shown). We presumed that volatilization of acids along with defrosting in winter resulted in acid loss. The ash concentration increased with elevated acidity, implying that the acidic conditions enhanced the dissolution of minerals in the vinegar. Interestingly, the concentration on either wet or dry mass basis of crude proteins (mostly amino acids and short peptides) and soluble sugar increased in the vinegar with moisture loss (Table 1), which provides opportunities for further generation of Maillard reaction products.

**Table 1:** The proximate composition of vinegar during ageing

Parameter	*t*(ageing)/month			
0	6	12	18
*γ*(moisture)/(g/L)	(873.5±14.1)^a^	(811.4±23.3)^b^	(723.8±19.8)^c^	(676.5±8.7)^d^
pH	(3.42±0.04)^a^	(3.54±0.10)^a^	(3.29±0.03)^b^	(3.08±0.02)^c^
*γ*(titratable acidity)/(g/L)	(45.5±4.4)^a^	(65.1±7.3)^b^	(78.8±2.2)^c^	(83.4±5.6)^d^
*γ*(crude protein)/(g/L)	(22.8±2.3)^a^	(37.5±3.7)^b^	(55.4±3.0)^c^	(67.5±8.8)^d^
*γ*(soluble sugar)/(g/L)	(11.6±0.6)^a^	(15.2±0.4)^b^	(26.8±0.7)^c^	(34.9±0.2)^d^
*γ*(ash)/(g/L)	(18.9±2.5)^a^	(31.7±0.6)^b^	(47.8±1.1)^c^	(54.5±3.1)^d^

The values represent mean±standard deviation (*N*=3). Mean values with different letters in superscript within the same column suggest significant differences at p<0.05

### Determination of Maillard reaction product concentrations in aged sorghum vinegar with HPLC analysis

**Fig. 1:**
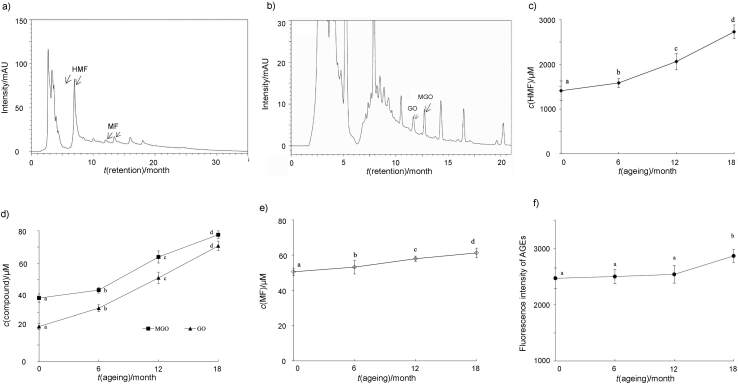
Analysis of 5-hydroxymethylfurfural (HMF), 5-methylfurfural (MF), methylglyoxal (MGO), glyoxal (GO) and advanced glycation end-products (AGEs) in ageing vinegar: a) representative HMF and MF peaks detected in the HPLC chromatograms of vinegar at *λ*=283 nm, b) representative MGO and GO peaks in the HPLC chromatograms of vinegar at *λ*=316 nm, c) changes in HMF concentration, d) changes in MGO and GO concentrations, e) changes in MF concentration, and f) changes in the fluorescence intensity of AGEs

HPLC method was used to determine the concentration of Maillard reaction products (HMF, MF, MGO and GO) in ageing vinegar. Fig. 1a and Fig. 1b show the HMF (6.965 min retention time) and MF (13.417 min) peaks, and MGO (12.722 min) and GO (11.668 min) peaks in representative chromatograms of aged sorghum vinegar samples, respectively, while Fig. 1c, Fig. 1d and Fig. 1e indicate the changes in their concentration during the ageing of vinegar. Initially, the concentrations of HMF and MF were 1409.7 and 50.47 μM, respectively, while of MGO and GO were 38.7 and 21.4 μM, respectively, which is consistent with the ranges reported for the final Maillard reaction products of vinegar, *i.e.* 185–1670 μM for HMF, 4.52–71.56 μM for MF, 31.9–61.6 μM for MGO, and 10–85 μM for GO (*1*). It is well known that the primary purpose of fuming of aged sorghum vinegar, in which vinegar is heated at 80–90 °C for 6 days, results in a reddish-brown colour and caramel flavour due to the Maillard reaction. HMF and MF are furans obtained *via* direct hydrolyzation of sugar under acidic conditions (*35*). MGO and GO, both key reactive dicarbonyl species, also tend to form *via* Maillard reaction and monosaccharide autooxidation (*36*). Therefore, it is no surprise that in this study these Maillard reaction products in aged sorghum vinegar have high concentration immediately after fuming. During the 18-month ageing in this study, concentration of all Maillard reaction products, in particular HMF, MGO and GO, increased significantly. Specifically, HMF, MGO and GO concentration increased slowly in the first six months, and peaked rapidly in the last 12 months. At the end of ageing, their concentrations were 2725.4, 77.5 and 70.7 μM respectively, which was 1.9-, 2.0- and 3.3-fold increase, respectively, compared with the starting concentration. However, the MF concentration increased only by 1.2-fold. This result is consistent with the trends in total sugar and protein concentrations indicated above and thus suggests that the ageing increased the acidity, protein and sugar amount, which promoted the formation of Maillard reaction products. Currently, according to the European standard, the HMF of honey is less than 450 μM (*1*). It is clear that HMF in aged sorghum vinegar exceeded this limit. Although the concentrations of MGO and GO intake in foods are not established, the effects of their long-term consumption have attracted significant attention. Therefore, due to the continuous increase in Maillard reaction product concentrations in aged sorghum vinegar during ageing in this study, it is important to reduce the ageing time of vinegar without compromising the flavour and colour.

The amount of AGEs is also controlled in foods due to their close relationship with diseases such as diabetes and Alzheimer (*37*). High concentrations of sugar and protein together with high temperature result in the formation of AGEs (*38*). In this study, the evolution of AGEs during vinegar ageing was also observed *via* fluorescent analysis (Fig. 1f). No apparent changes in the amounts of AGEs were detected during the first 12 months of ageing; however, a significant increase was detected at the end of the process. Generally, the formation of AGEs requires a slightly alkaline environment (*39*). The highly acidic environment was clearly unfavourable for the formation of AGEs during the first 12 months of ageing in this study. However, even though the acidity increased in the last six months, the continuous increase in the concentrations of sugar and protein in vinegar enhanced the formation of AGEs. This result further explains the importance of reducing the time of ageing during the production of aged sorghum vinegar.

Melanoidins are the brown nitrogenous polymers and copolymers generated *via* interaction between reducing sugars and compounds carrying free amino groups during the advanced phase of Maillard reaction (*2*). The generation of melanoidins often results in toasty flavour and increased antioxidant activity. Their concentrations in aged sorghum vinegar in this study were 10.2, 16.0, 24.4 and 29.7 mg/mL at 0, 6, 12 and 18 months, respectively (data not shown). This increasing trend is consistent with the reduction in moisture, and the increased acidity and protein and sugar concentration. Further assay revealed melanoidin growth rates of 56.9% (0–6 months), 52.5% (6–12 months) and 21.7% (12–18 months) (data not shown), suggesting that their concentration increased predominantly in the first 12 months of ageing. The elevated acidity might adversely impact the formation of Maillard reaction products during the advanced phase of Maillard reaction. Currently, the ageing of sorghum vinegar generally lasts from 1 to 3 years, and sometimes exceeds 10 years. Our assays of intermediate- and advanced-phase Maillard reaction products revealed that reducing the ageing time is essential to ensure the safety and functionality of food products.

### Proximate composition and physicochemical properties of vinegar melanoidin

**Fig. 2:**
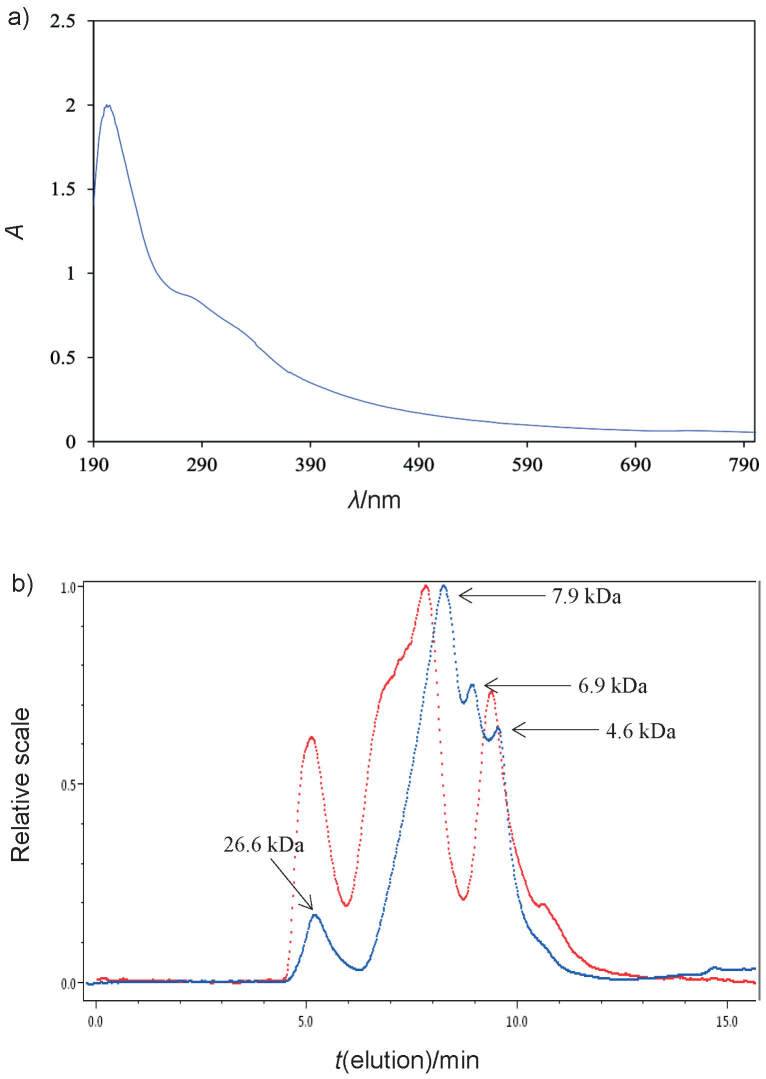
The profile of vinegar melanoidin: a) UV/Vis spectrum and b) high-performance gel permeation chromatography. The red and blue curves represent the results of analysis with a light scattering detector and refractive index detector, respectively

In this study, vinegar melanoidin was derived from the 18-month aged sorghum vinegar with a yield of 18.2 mg/mL (data not shown), which was mainly a pure melanoidin skeleton generated by the loss of non-covalently bound compounds following NaCl treatment. The absorption coefficient of vinegar melanoidin was *K*_420 nm_=(0.64±0.02) L/(cm·g) (data not shown), and its UV-Vis spectrum resembled that of melanoidins reported previously (*14*), in which the peak exhibited featureless end absorption but increased in intensity at lower wavelength (Fig. 2a). Therefore, vinegar melanoidin was mainly composed of pure melanoidins. HPGPC showed that vinegar melanoidin was a non-homogenous melanoidin with four peaks at *M*_r_=26.6, 7.9, 6.9 and 4.6 kDa (Fig. 2b), indicating its complex composition. Proximate analysis revealed that vinegar melanoidin contained (322±28) mg/g of protein and (429±22) mg/g of soluble sugar (data not shown), suggesting that carbohydrates and proteins were the major constituents of the melanoidin in the aged sorghum vinegar. Further assays also revealed that vinegar melanoidin contained (7.6±1.6) mg/g of phenolics (data not shown), substantially less than the phenolic mass fractions of pure melanoidin skeleton in the vinegar reported previously (*10*). These low-level irremovable phenolic compounds might participate in the Maillard reaction during fuming and thereby become incorporated into vinegar melanoidins (*14*). In addition, only trace or undetectable amounts of HMF, MF, MGO and GO were found in the HPLC spectra (data not shown). These results suggested that ultrafiltration and saline treatment effectively removed non-covalently bound compounds, resulting in vinegar melanoidin, which is a pure melanoidin. Thus, it is appropriate to use it to analyze the properties of melanoidin skeleton.

**Fig. 3:**
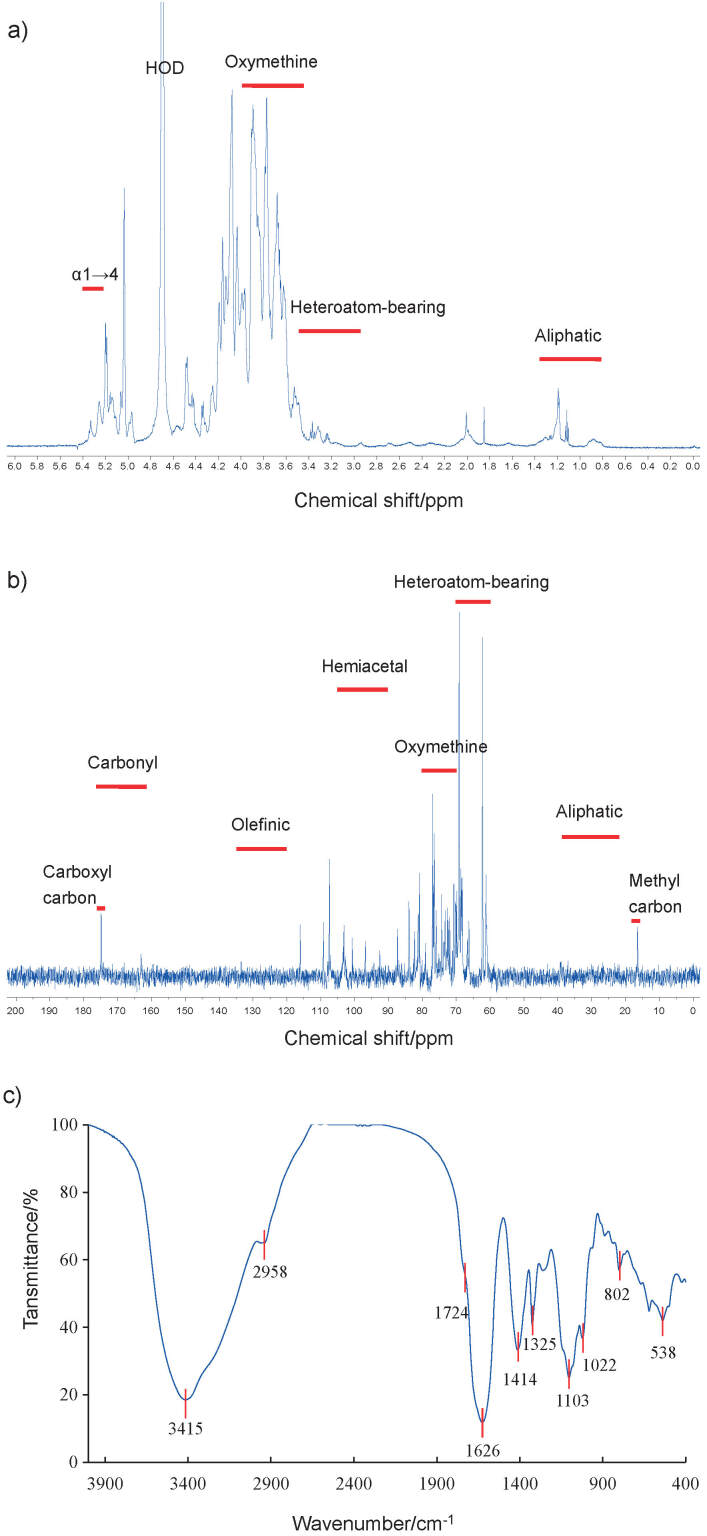
Vinegar melanoidin spectra: a) ^1^H, b) ^13^C NMR and c) FTIR. HOD=deuteroxide

NMR analysis was conducted to obtain additional insights into the structure of vinegar melanoidin (Fig. 3a and Fig. 3b). The carbohydrate moiety showed broad signals of oxymethine (3.4–4.0 ppm in ^1^H NMR and 70–80 ppm in ^13^C NMR) and hemiacetal signals (4.5–5.4 ppm in ^1^H NMR and 90–105 ppm in ^13^C NMR) (*40*). The non-sugar moieties in vinegar melanoidin were also detected under low magnetic fields at 120–180 ppm representing olefinic and carbonic functions, and high magnetic fields at less than 70 ppm indicating aliphatic and heteroatom-bearing carbons (*40*). In particular, the sharp signals at around 16 and 175 ppm can be assigned to methyl carbon and carboxyl carbon, respectively, both probably belonging to the amino acid residues in the melanoidin (*41*). These signals indicated that vinegar melanoidin was a typical melanoidin. The absorption of glycans was clearly seen in the IR spectrum (Fig. 3c), including the typical broad band of OH stretching vibration at 3415 cm^-1^, the characteristic absorption bands at 2958 cm^-1^ associated with CH_3_/CH_2_ stretching, a band at 1414 cm^-1^ associated with CH/CH_2_ bending vibration, and bands at 1200-1000 cm^-1^ attributed to OH stretching or C-O-C stretching due to glycosidic bonds. Non-sugar moieties were also observed in the IR spectrum, based on the sharp absorption at 1626 cm^-1^ attributed to carbonyl (aligning with the 170-175 ppm shifts in the ^13^C NMR spectrum), C=C or C=N double bond stretching (*40*), a weak shoulder at 1724 cm^-1^ due to either C=O or COOH, and an absorption at 1325 cm^-1^ for O-H bending or C-N stretching in the melanoidins composed of amino acids (*41*). In summary, O-H, C-H, COOH, C=O, C=C, C=N, N-N, C-O and C-N groups were present in the melanoidin skeleton, further establishing the presence of melanoidin-like components in the melanoidin skeleton of the vinegar.

### In vitro antioxidant activity of vinegar melanoidin

The *in vitro* antioxidant activity of vinegar melanoidin was assayed before analyzing its *in vivo* hepatoprotective properties. Vinegar melanoidin demonstrated strong scavenging activity against DPPH radicals with an IC_50_ value, as Trolox equivalents, of (30.2±2.6) mg/g, and an oxygen radical absorbance capacity (ORAC) expressed as Trolox molal concentration of (218.5±18.1) μmol/g (data not shown). These amounts are comparable to those of high-molecular-mass melanoidins in Zhenjiang rice vinegar (*22*). It is well known that melanoidins derived from various sources exhibit antioxidant activity, but most studies attributed their antioxidant activity in vinegar to the phenolics bound to the melanoidin skeleton. In this study, the amount of phenolics in vinegar melanoidins was as low as 0.76%. However, the pure vinegar melanoidin showed strong radical-scavenging activity and disrupted the free radical chain reaction. This action of vinegar melanoidin against free radicals might be attributed to the hydroxyl groups as well as a small amount of phenolics in the melanoidin skeleton (*42*).

### Serum and hepatic biochemical parameters

CCl_4_ generates free radicals following metabolism by hepatic microsomes. These free radicals induce lipid peroxidation of hepatocellular membrane and subsequent necrosis of hepatocytes, leading to pathological changes similar to those observed in acute viral hepatitis (*43*), drug/chemicals-induced hepatopathy and oxidative stress (*13*). The increase in transaminase amounts in serum and hepatic MDA concentration is a major indication of diminished liver function. In this study, the values of AST, ALT, ALP, total bilirubin and lipid peroxidation in hepatic cells were significantly elevated in the CCl_4_-treated rats (Table 2), indicating that CCl_4_ treatment resulted in the loss of functional integrity of hepatic cell membrane and hepatic cellular leakage (*44*). Feeding the CCl_4_-treated rats with vinegar melanoidin at 100, 200 and 400 mg/kg significantly reduced their serum AST, ALT, ALP and total bilirubin activities, in a dose-dependent manner. The effect of vinegar melanoidin on serum and hepatic biochemical parameters of CCl_4_-treated rats was similar or even greater than that of silymarin, which was used as a hepatoprotective control drug in this study. In particular, the effects of 200 and 400 mg/kg vinegar melanoidin on the decrease of the serum glutamic oxaloacetic transaminase (GOT) and glutamic pyruvic transaminase (GPT) were more prominent than those of silymarin (100 mg/kg). Meanwhile, vinegar melanoidin also significantly decreased CCl_4_-induced hepatic lipid peroxidation, as demonstrated by the lower concentrations of MDA in the liver homogenates of rats fed vinegar melanoidin (p<0.05). These results suggested that vinegar melanoidin inhibit the CCl_4_-induced hepatic damage in rats based on the hepatic detoxification of xenobiotics and substantial normalization of serum transaminase levels, and stabilization of plasma membranes and functional integrity of the cell membrane.

**Table 2:** Effects of vinegar melanoidin (VMD) administration on the serum and hepatic biochemical parameters of rats

Group	Serum enzyme activity				Liver index	
AST/(U/L)	ALT/(U/L)	ALP/(U/L)	*γ*(bilirubin in blood)/(mg/100 mL)	*b*(MDA)/(nmol/mg)	*b*(GSH)/(µmol/mg)
Control	(238±19)	(104±12)	(449±76)	(0.10±0.01)	(2.54±0.29)	(3.3±0.5)
CCl_4_	(4216±1174)^###^	(2533±1041)^###^	(1084±211)^###^	(0.43±0.12)^###^	(3.44±0.23)^###^	(2.3±0.4)^###^
VMD_100_+CCl_4_	(438±216)***	(382±193)***	(720±226)*	(0.32±0.13)	(2.68±0.05)*	(2.8±0.5)*
VMD_200_+CCl_4_	(351±182)***	(249±141)***	(593±124)**	(0.21±0.07)*	(2.11±0.09)**	(3.2±0.3)**
VMD_400_+CCl_4_	(225±87)***	(223±98)***	(551±131)**	(0.16±0.04)**	(1.89±0.15)**	(3.5±0.3)**
Silymarin_100_+CCl_4_	(426±173)***	(434±158)***	(581±185)**	(0.15±0.04)**	(2.04±0.13)**	(3.3±0.6)**

Index 100, 200 and 400 denotes mass fraction in mg/kg. AST=aspartate transaminase, ALT=alanine transaminase, ALP=alkaline phosphatase, MDA=malondialdehyde, GSH=glutathione. The values are expressed as mean±standard deviation (*N*=3). ^###^p<0.005, when compared with control group, *p<0.05, **p<0.01, ***p<0.005, when compared with *φ*(CCl_4_, water)=20% treated group

**Fig. 4:**
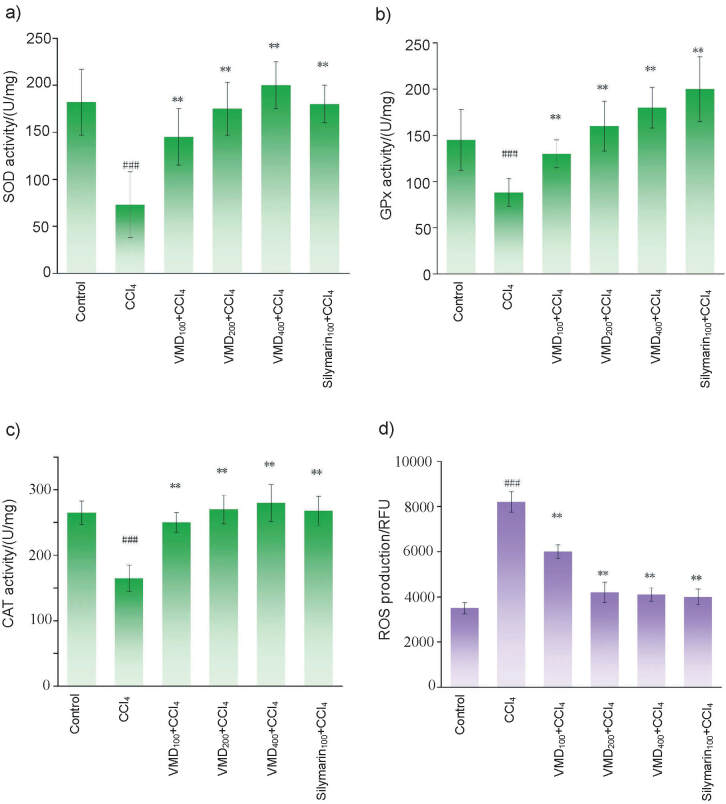
Effects of vinegar melanoidin (VMD) on the activities of: a) superoxide dismutase (SOD), b) glutathione peroxidase (GPx), c) catalase (CAT), and d) generation of reactive oxidative species (ROS) in the rat liver. RFU=relative fluorescence units. Index 100, 200 and 400 denotes mass fractions in mg/kg. *φ*(CCl_4_, water)=20%. The data are presented as mean value±S.D., *N*=8. ^###^p<0.05, compared with the control group, **p<0.05, compared with the group treated only with 20% CCl_4_

Most of the antioxidants and antioxidant enzymes in the liver, including glutathione (GSH), SOD, GPx and CAT, sequester ROS and/or maintain cells and cellular components in the appropriate redox state, and thus constitute the first line of defense against free radicals. In this study, the toxicity of CCl_4_ significantly decreased the hepatic GSH concentration in protein from (3.3±0.5) in the control group to (2.3±0.4) μmol/mg (Table 2), whereas the addition of silymarin and vinegar melanoidin at dietary doses of 100 g/kg reversed the GSH concentration. The high doses of vinegar melanoidin (200 and 400 mg/kg) restored the GSH almost completely to the normal concentration in the CCl_4_-treated rats. CCl_4_ treatment significantly decreased the activities of SOD, GPx and CAT in the rat liver while vinegar melanoidin supplementation significantly restored the activities of SOD, GPx and CAT in CCl_4_-treated rats (p<0.05) in a dose-dependent manner (Fig. 4a, Fig. 4b and Fig. 4c). The effectiveness of 200 and 400 mg/kg vinegar melanoidin in recovering antioxidant enzymes in the rat liver was similar to that of silymarin (100 mg/kg). These results further confirmed the hepatoprotective action of the melanoidin by restoring the antioxidant properties and antioxidant enzyme activities.

The direct influence of vinegar melanoidin on suppressing ROS was further investigated. Fig. 4d shows that the highest amounts of ROS were found in the liver of rats treated solely with CCl_4_ (p<0.05). The addition of vinegar melanoidin to rat diet significantly reduced the formation of ROS. The suppression of ROS in rat livers treated with 200 and 400 mg/kg vinegar melanoidin was close to that of 100 mg/kg silymarin, which was consistent with the results of *in vitro* antioxidant and MDA analysis. These findings suggested that vinegar melanoidin directly inhibited the generation of ROS *in vivo* and their free-radical scavenging activity facilitated the antioxidant effects of enzymes in the liver.

### Histological analysis

**Fig. 5:**
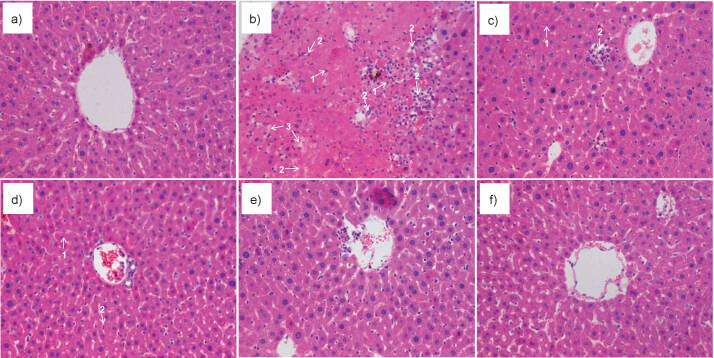
Photomicrographs (×40) of liver sections obtained from: a) control rats, b) rats treated with CCl_4_, c) rats treated with CCl_4_ and *w*(silymarin)=100 mg/kg, and d-f) rats treated with CCl_4_ and *w*(vinegar melanoidin)=100 (low), 200 (medium) and 400 mg/kg (high) respectively. Liver sections were stained with hematoxylin and eosin. 1=hepatic cell necrosis and apoptosis, 2=inflammatory cell infiltration, 3=hepatic sinus expansion and edema

Microscopic examination of the liver sections in the control group showed normal histological features, and the hepatic lobules showed a clear structure and ordered cells (Fig. 5a). Feeding the rats (control group) with CCl_4_ alone severely damaged the liver. The hepatic lobules lost their clear structure, specifically due to hepatocyte necrosis, apoptosis and vacuolar degeneration, and inflammatory cell infiltration (Fig. 5b). Feeding 100 mg/kg of silymarin partially protected the rats from the harmful hepatic effects of CCl_4_. Moderate vacuolar degeneration and mild hepatocyte necrosis, apoptosis and inflammatory cell infiltration are observed in the micrographs of silymarin-treated rat livers (Fig. 5c). However, the presence of vinegar melanoidin in the diet dose-independently reduced hepatocyte necrosis or apoptosis, vacuolar degeneration and macrophage infiltration (Fig. 5d, Fig. 5e and Fig. 5f). Specifically, treatment with 400 mg/kg vinegar melanoidin was the most effective in protecting the rat liver against the hepatotoxic effects of CCl_4_ (Fig. 5f). These findings are consistent with those of biological parameters above, suggesting that vinegar melanoidin significantly ameliorated CCl_4_-induced liver injury, so it can be used for liver protection.

## CONCLUSIONS

This study demonstrated that prolonged ageing increases the amounts of unhealthy intermediate Maillard reaction products in the aged sorghum vinegar, such as 5-hydroxymethylfurfural, 5-methylfurfural, methylglyoxal, glyoxal and advanced glycation end products. Their concentrations increased rapidly during the late stage of ageing. Therefore, appropriate reduction in ageing duration is essential for practical aged sorghum vinegar production to balance safety and sensory properties. This study also established the protective effects of pure vinegar melanoidins and advanced phase Maillard reaction products on oxidative stress-induced liver damage *in vitro* and *in vivo*. The biochemical ingredients in pure vinegar identified *via* histopathological analysis exhibited potent hepatoprotective activity, which was mediated by the suppression of the formation of hepatic ROS and restoration of the activities of antioxidant enzymes, which may represent a promising preventive strategy against liver injury. These findings presented novel insights into the generation progress of major vinegar Maillard reaction products during ageing, in particular the *in vivo* protection by melanoidin against hepatic oxidative damage beyond the previous *in vitro* investigations, advancing our understanding of the generation and biological activity of Maillard reaction products from vinegar.
